# Supplementary material for: Transcription factors ASCL1 and OLIG2 drive glioblastoma initiation and co-regulate tumor cell types and migration
Source: Nat Commun. 2024 Nov 28;15:10363. doi: 10.1038/s41467-024-54750-9 (PMC11605073; doi:10.1038/s41467-024-54750-9)
Supplement: Supplementary file 1 — Supplementary Information [file 41467_2024_54750_MOESM1_ESM.pdf]

SUPPLEMENTARY FIGURES & LEGENDS

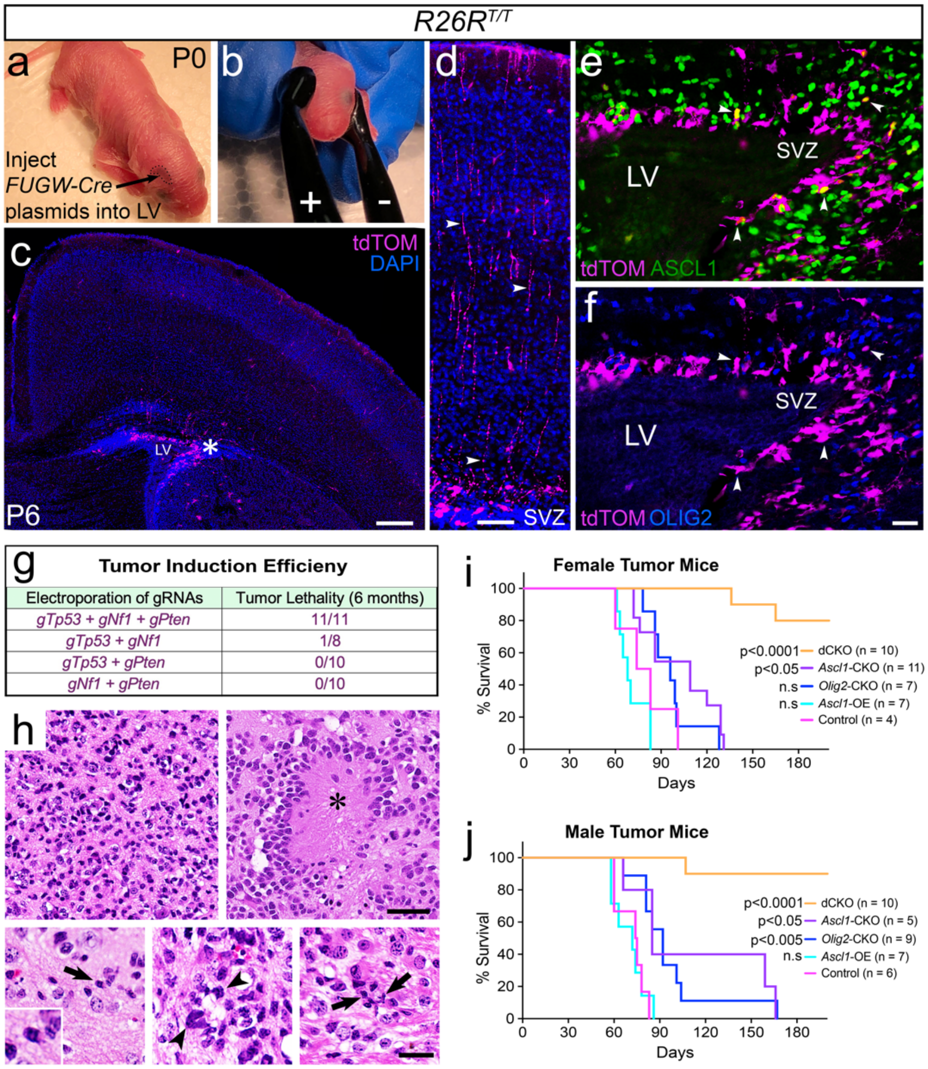

Supplementary Fig. 1. Electroporation of NPCs in the SVZ of *R26R$^{T/T}$* mice. (**a**,**b**) Mouse pups are injected with *FUGW-Cre* plasmids into right lateral ventricle (LV; dotted line in Panel **a**) and electroporated at P0. (**c**,**d**) tdTOM and DAPI staining at P6 showing Cre-mediated labeling of radial glia in the dorsal SVZ with radial processes extending to the pia surface (arrowheads). Asterisk marks region imaged for panels E and F. (**e**,**f**) Only a few tdTOM+ cells express ASCL1 and OLIG2 (arrowheads). These results were observed in n=3 animals of several liters. (**g**) Table demonstrating tumor incidence with electroporation of all three or combinations of two gRNA plasmids targeting *Tp53, Nf1*, and *Pten*. (**h**) H&E staining of control tumors at terminal stages demonstrating hypercellularity within tumor regions (top left panel), pseudopalisading necrosis (top right, asterisk), mitotic cells (bottom left, arrow), multinucleated giant cells (bottom middle, arrowhead), and nuclear atypia (bottom right, arrow). (**i**,**j**) Kaplan-Meier survival curve of female and male tumor mice for the various tumor types. Note that there is no significant difference in survival between male and female of the same tumor type, but only between control and the indicated experimental tumor groups (Mantel-Cox test). Scale bars: 250 μm for panel C, 100 μm for panel **d**, and 25 μm for panels E,F. For H, scale bars: 50 μm for top panels; 20μm for lower panels; 10μm for inset.

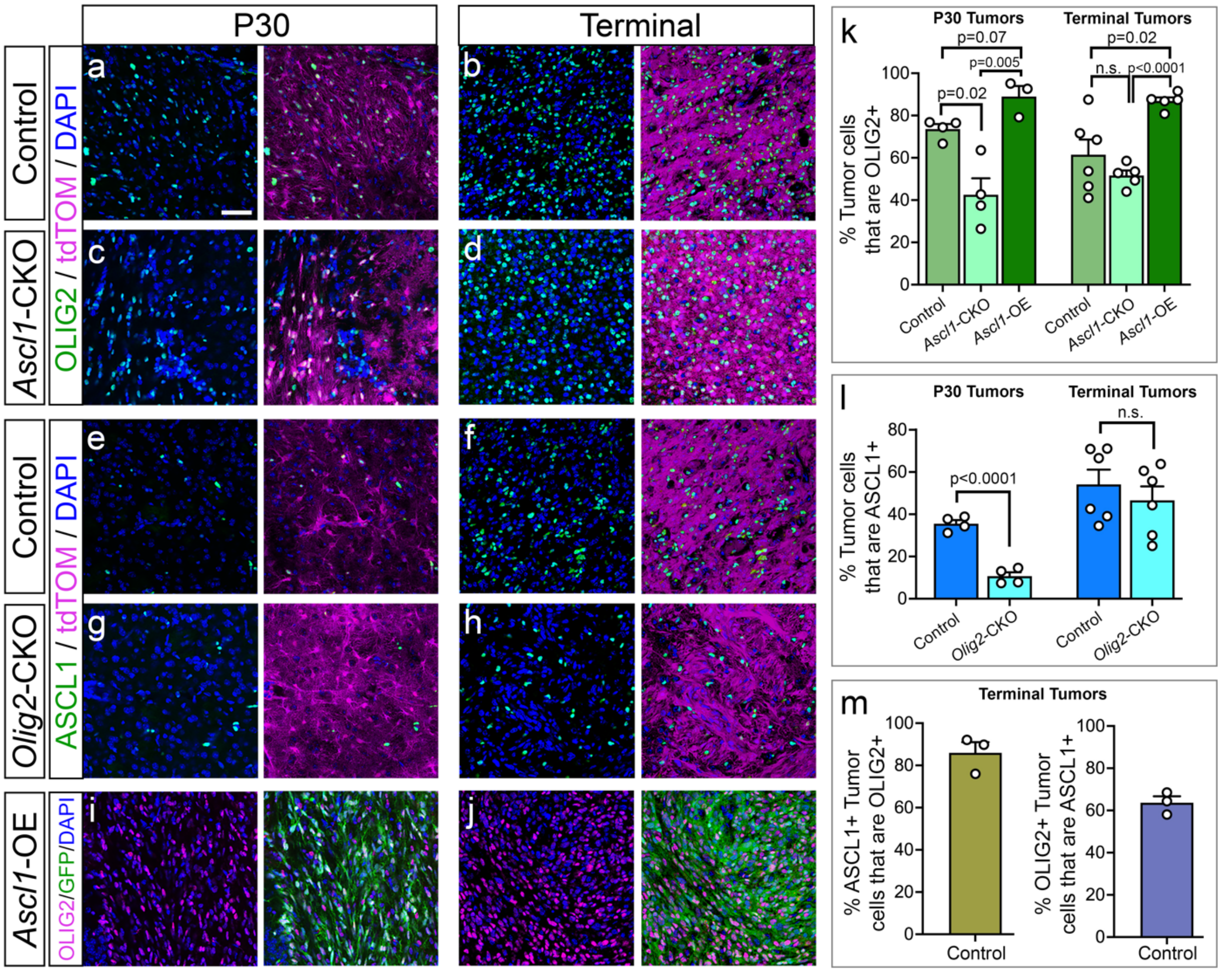

Supplementary Fig. 2. ASCL1 and OLIG2 reciprocally regulate expression in glioma tumors. (**a-j**) Representative immunofluorescent images of OLIG2+ tumor cells in control (**a,b**), *Ascl1*-CKO (**c,d**), and *Ascl1*-OE (I,j) tumors, or ASCL1+ tumor cells in control (**e,f**) and *Olig2*-CKO (g,h) tumors at P30 and terminal stages. (**k,l**) Percentage of labeled tumor cells positive for OLIG2 or ASCL1 for the different tumor types. **k**: P30 - control n = 4 mice, *Ascl1*-CKO n = 4 mice, *Ascl1*-OE n = 3 mice, Terminal - control n = 6 mice, *Ascl1*-CKO n = 5 mice, *Ascl1*-OE n = 5 mice; **l**: P30 n = 4 mice/genotype, Terminal n= 6 mice/genotype. (**m**) Percentage of ASCL1+ labeled tumor cells that are OLIG2+ and percentage of OLIG2+ labeled tumor cells that are ASCL1+. Data shown are mean ± SEM (n=3). Statistical significance was determined using unpaired t-tests with Welch's correction. Scale bar: 50 μm for all images. Source data are provided as a Source Data File.

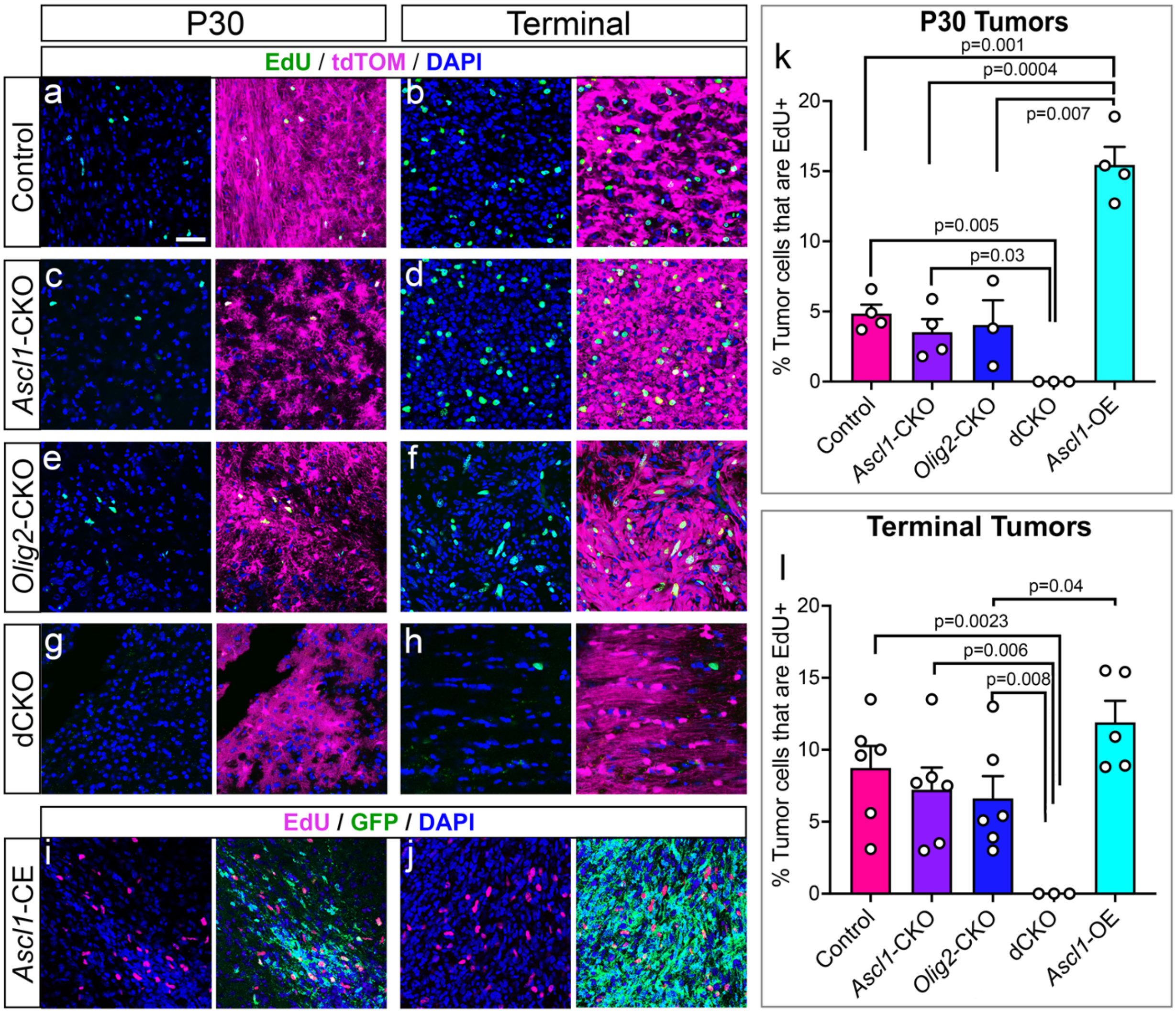

Supplementary Fig. 3. ASCL1 and OLIG2 are required for proliferation of brain tumors. **(a-j)** Representative images of EdU staining for control (**a,b**), *Ascl1*-CKO (C,D), *Olig2*-CKO (**e,f**), dCKO (**g,h**), and *Ascl1*-OE (i,j) tumors at P30 and terminal stages. **(k,l)** Percentage of tumor cells positive for EdU at P30 and terminal stages. Data shown are mean ± SEM. Open circle within bar graphs indicate the number of tumor mice analyzed for each genotype. Statistical significance was determined using unpaired t-tests with Welch's correction. k: Control n = 4 mice, *Ascl1*-CKO n = 4 mice, *Olig2*-CKO n = 3 mice, dCKO n = 3 mice, *Ascl1*-OE n = 4 mice; L: control n = 6 mice, *Ascl1*-CKO n = 6 mice, *Olig2*-CKO n = 6 mice, dCKO n = 5 mice, *Ascl1*-OE n = 5 mice. Scale bar: 50 μm for all images. Source data are provided as a Source Data File.

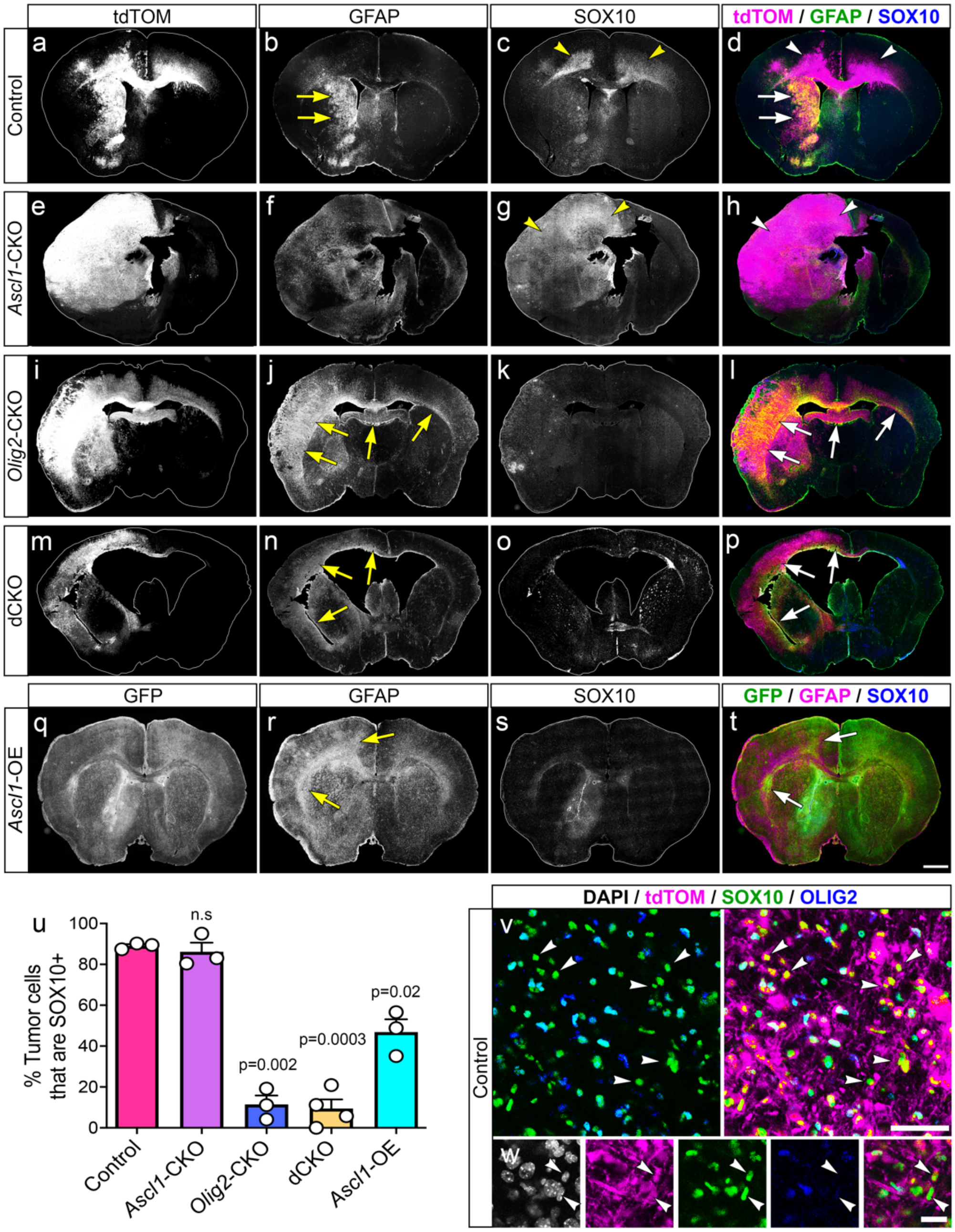

Supplementary Fig. 4. ASCL1 and OLIG2 regulate opposing tumor cell types.
(**a-t**) Whole brain fluorescent images demonstrating overlap of tumor reporter with either GFAP (arrows) or SOX10 (arrowheads) in control (**a-d**), *Ascl1*-CKO (**e-h**), *Olig2*-CKO (**i-l**), dCKO (**m-p**), and *Ascl1*-OE (Q-T) tumors. (**u**) Percentage of tumor cells positive for SOX10 at terminal stages. Data shown are mean ± SEM. Open circle within bar graphs indicate the number of tumor mice analyzed for each genotype. Statistical significance was determined using unpaired t-tests with Welch's correction compared to control. Control n = 3 mice, *Ascl1*-CKO n = 3 mice, *Olig2*-CKO n = 3 mice, dCKO n = 4 mice, *Ascl1*-OE n = 3 mice. (**v,w**) Double immunofluorescence of SOX10 and OLIG2 in terminal control tumor. Arrowheads indicate SOX10+;tdTOM+ tumor cells that are OLIG2-. Scale bar: 1mm for A-T, 50 μm for V, and 25 μm for W. Source data are provided as a Source Data File.

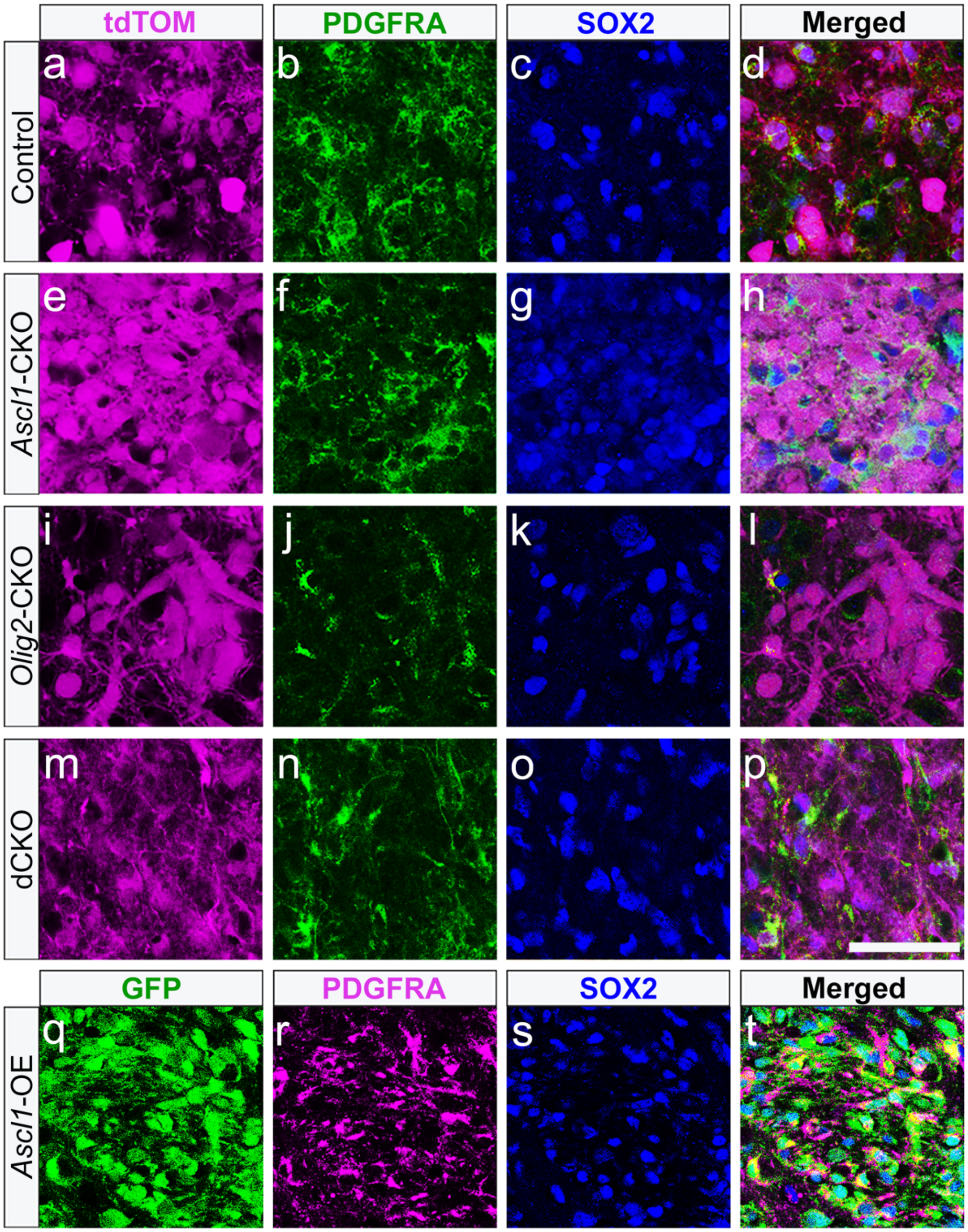

Supplementary Fig. 5. SOX2 and PDGFRA are differentially expressed in mouse brain tumors. (**a-t**) Double immunofluorescence showing that SOX2 is expressed in tumor cells of all tumor types whereas PDGFRA expression is high in control (**a-d**), *Ascl1*-CKO (**e-h**), and *Ascl1*-OE (**q-t**) tumors, but low in *Olig2*-CKO (I-L) and dCKO (**m-p**) tumors. Results were observed in n=3 mice/genotype. Scale bar: 25 μm for all images.

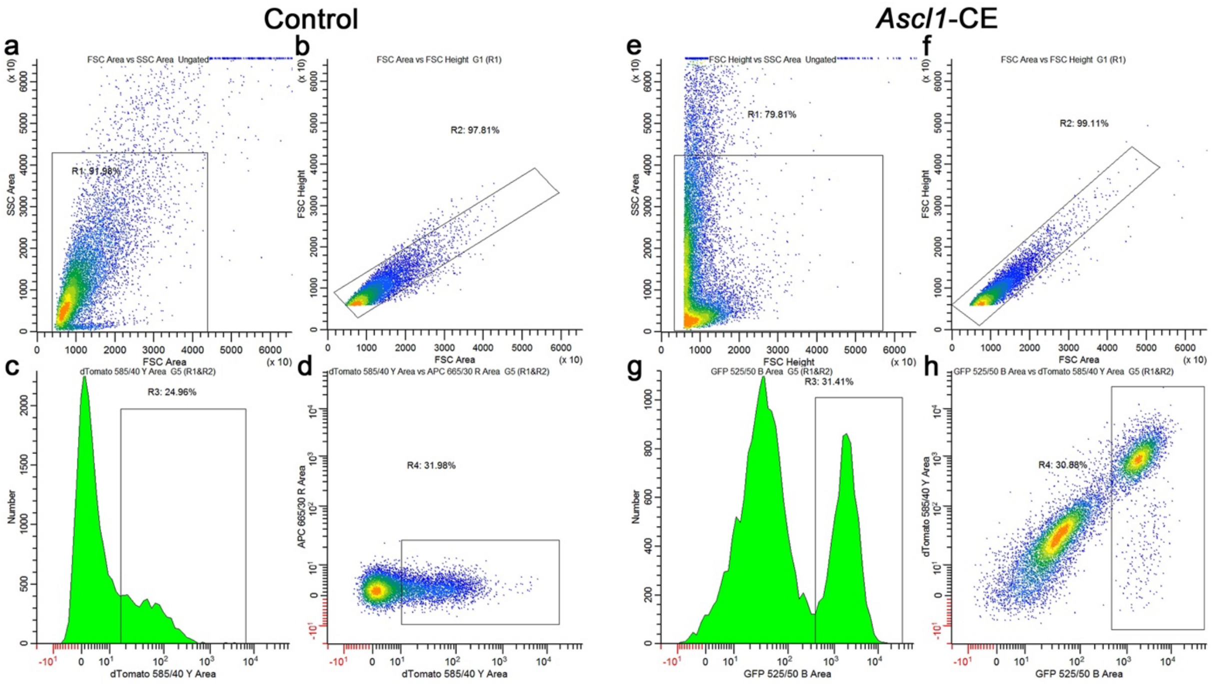

Supplementary Fig. 6. Gating for FAC sorting of viable control (tdTOM+) and *Ascl1*-OE (GFP+) tumor cells. (**a-d**) Representative gating strategy for isolation of tdTom+ control tumor cells. (**e-h**) Representative gating strategy for isolation of tdTom+GFP+ *Ascl1*-OE tumor cells. (**a,e**) Isolation of cells based on cell size and complexity/granularity. FSC (Forward scatter): cell size; SSC (side scatter): granularity. (**b,f**) Isolation of singlets. (**c,d**) Isolation of tdTom+ control tumor cells. (**g,h**) isolation of tdTom+GFP+ *Ascl1*-OE tumor cells. Note: *Ascl1*-OE tumor mice were crossed with an *R26-tdTomato* line to make tumor cells visible for dissection and subsequent dissociation.

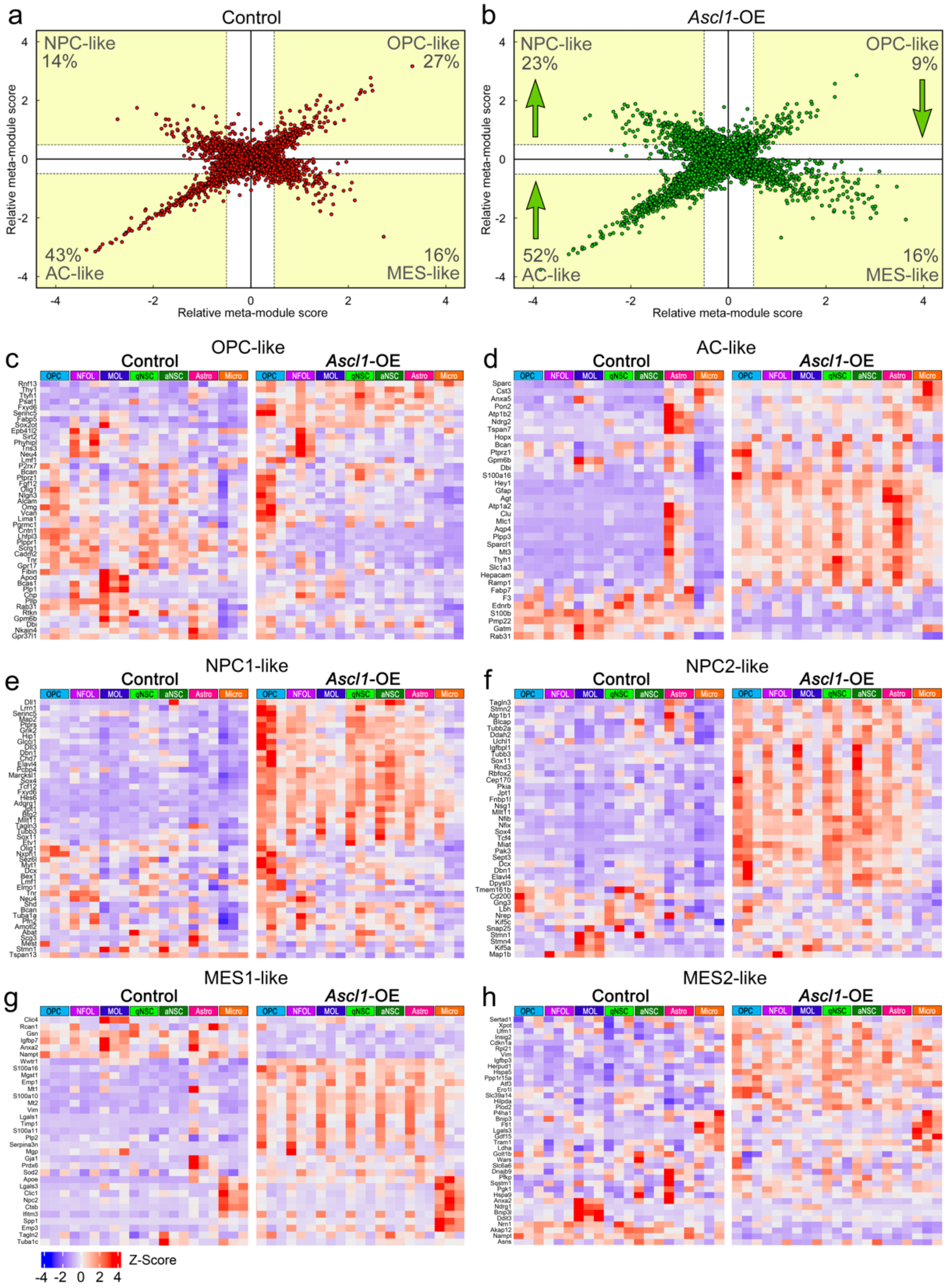

Supplementary Fig. 7. NPC-like and AC-like cell states are enriched in *Ascl1*-OE tumor cells. (**a,b**) Neftel et al (2019) GBM cellular state assignment of control (**a**) and *Ascl1*-OE (**b**) tumor cells. Percentage represents proportion of high-scoring cells for the indicated cellular states showing an increase in NPC-like and AC-like cells and a decrease in OPC-like cells (green arrows). (**c-h**) Heatmap of Unionized Cell Type RNA-seq for OPC-like (**c**), AC-like (**d**), NPC1- and NPC2-like (**e,f**), and MES1- and MES2-like (**g,h**) signature genes in control and *Ascl1*-OE tumors.

SUPPLEMENTARY TABLES

Supplementary Table 1

Mouse Reagents	Sources
R548 & R738 PDOX-GBM	Dr. Robert M. Bachoo; Vue et al, 2018. Glia 68, 2613-2630
Rosa26-LSL-tdTomato (*R26R$^{T/T}$*)	JAX:007914; Madisen et al, 2010. Nature Neuroscience 13, 133-140
Ascl1^{floxed} (*Ascl1$^{F/F}$*)	Dr. Francois Guillemot, via Dr. Jane E. Johnson; Pacary et al, 2011. Neuron 69, 1069 – 1084
Olig2^{floxed} (*Olig2$^{F/F}$*)	Dr. Q. Richard Lu; Yue et al, 2006. Journal of Neuroscience 1275-80
Rosa26-LSL-tTA (*R26R$^{tTA/tTA}$*)	JAX:008603; Wang et al, 2008. Neurobiology of Disease 29(3):400-8
TetO-Ascl1-ires-GFP (*TetO-AIG*)	Dr. Masato Nakafuku, via Dr. Thomas A. Reh; Ueki et al, 2015. PNAS 112(44), 13717 – 13722

Supplementary Table 2

Antibodies and Research Reagents	Sources
Guinea Pig anti-ASCL1 (1:1000)	Johnson Lab (TX518); Kim et al, 2008 [116]
Mouse anti-ASCL1 (1:1000)	BD Biosciences (556604)
Rabbit anti-ASCL1 (1:1000)	Abcam (ab211327)
Rabbit anti-GFAP (1:500)	Millipore (AB5804)
Mouse anti-OLIG2 (1:250)	Millipore (MABN50)
Rabbit anti-OLIG2 (1:1000)	Millipore (AB9610)
Rat anti-PDGFRa (1:100)	BD Pharmigen (558774)
Rabbit anti-SOX2 (1:500)	Millipore (AB5603)
Goat anti-SOX10 (1:50)	R&D Systems (AF2864)
Donkey anti-goat 488 (1:500)	Invitrogen (A11055)
Donkey anti-goat 568 (1:500)	Invitrogen (A11057)
Donkey anti-mouse 488 (1:500)	Invitrogen (A21202)
Donkey anti-mouse 568 (1:500)	Invitrogen (A10037)
Donkey anti-rabbit 488 (1:500)	Invitrogen (A21206)
Donkey anti-rabbit 647 (1:250)	Invitrogen (A31573)
Donkey anti-rat 647 (1:250)	Invitrogen (A78947)
Goat anti-mouse 488 (1:500)	Invitrogen (A11001)
Goat anti-mouse 568 (1:500)	Invitrogen (A11004)
Goat anti-rabbit 488 (1:500)	Invitrogen (A11008)
Goat anti-rabbit 568 (1:500)	Invitrogen (A11011)
Goat anti-rabbit 647 (1:250)	Invitrogen (A21244)
Goat anti-rat 647 (1:250)	Invitrogen (A21247)
Normal Donkey Serum	Jackson Immuno Research Laboratories (017-000-121)
Normal Goat Serum	Jackson Immuno Research Laboratories (005-000-121)
Neural Tissue Dissociation Kit (P)	Miltenyi Biotec (130-092-628)
5-ethynyl-2′-deoxyuridine (EdU)	BIONSYNTH (NE08701)
Sulfo-Cyanine5 azide	Lumiprobe (A3330)
