# Supplementary material for: Transcription factors ASCL1 and OLIG2 drive glioblastoma initiation and co-regulate tumor cell types and migration
Source: Nat Commun. 2024 Nov 28;15:10363. doi: 10.1038/s41467-024-54750-9 (PMC11605073; doi:10.1038/s41467-024-54750-9)
Supplement: Supplementary file 2 — Description of Additional Supplementary Files [file 41467_2024_54750_MOESM2_ESM.pdf]

Description of Additional Supplementary Files

Supplementary Data 1. Statistically significant ASCL1 and OLIG2 ChIP-seq binding peaks in PDOX-GBMs. Signal enriched regions following ChIP-seq of PDOX-GBMs (R548 and T738 cell lines) with anti-ASCL1 and anti-OLIG2 antibodies. "R548_ASCL1_Peaks": significant peaks from R548 ASCL1 ChIP-seq analysis. "T738_ASCL1_Peaks": significant peaks from T738 ASCL1 ChIP-seq analysis. "R548_OLIG2_Peaks": significant peaks from R548 OLIG2 ChIP-seq analysis. "T738_OLIG2_Peaks": significant peaks from T738 OLIG2 ChIP-seq analysis. Regions with enriched signal compared to input were identified using the fold-change over input and the poisson distribution.

Supplementary Data 2. Associated genes of ASCL1 and OLIG2 ChIP-seq binding peaks. Genomic Regions Enrichment of Annotations Tool (GREAT) assigned genes associated with significant binding peaks of ASCL1 ("13,457 ASCL1 Peaks-GenomicAssoc") and OLIG2 ("105,471 OLIG2 Peaks-GenomicAssoc") in PDOX-GBMs.

Supplementary Data 3. Genes with correlated expression to *ASCL1* in GBMs. Correlative expression analysis of TCGA GBM samples. Green: genes with positively correlated expression to *ASCL1*; Red: genes with negatively correlated expression ("GBM_levle3_tcga_cor_wrt_ASCL1.t"). Strength of association was determined using Spearman's rank correlation.

Supplementary Data 4. Genes with correlated expression to *OLIG2* in GBMs. Correlative expression analysis of TCGA GBM samples. Green: genes with positively correlated expression to *OLIG2*; Red: genes with negatively correlated expression. Strength of association was determined using Spearman's rank correlation.

Supplementary Data 5. Functional annotation of ASCL1 and OLIG2 target genes with positively correlated expression. DAVID Gene ontology of 841 ASCL1 and OLIG2 target genes derived from PDOX-GBMs with positively correlated expression to *ASCL1* and *OLIG2* in TCGA GBM samples using UP keywords. Statistical significance of pathway enrichment determined using Fisher's Exact test.

Supplementary Data 6. Functional annotation of ASCL1 and OLIG2 target genes with negatively correlated expression. DAVID Gene ontology of 211 ASCL1 and OLIG2 target genes derived from PDOX-GBMs with negatively correlated expression to *ASCL1* and *OLIG2* in TCGA GBM samples using UP keywords and the GO category Biological Processes (BP). Statistical significance of pathway enrichment determined using Fisher's Exact test.

Supplementary Data 7. Cell type gene signatures used to assign GBM cell type identities for scRNA-seq of control and Ascl1-OE tumor cells. Lineage-specific gene lists used for GBM cell type assignment in scRNA-seq analysis. Bolded gene names are targets of ASCL1.

Supplementary Data 8. Scaled unionized RNA-seq expression data by cell type for control and *Ascl1*-OE tumors. scRNA-seq gene expression was averaged by cell type for each control and *Ascl1*-OE tumor and then scaled to obtain z-scores for each gene.

Supplementary Data 9. Genes significantly differentially expressed between control and *Ascl1*-OE tumor cells. DEGs-Up: genes that are significantly upregulated in a cell type x cell type comparison of control and *Ascl1*-OE tumor cells. DEGs-Down: genes that are significantly downregulated in a cell type x cell type comparison of control and *Ascl1*-OE tumor cells. Statistical

significance of gene expression determined using Model-based Analysis of Single-cell Transcriptomics (MAST).

Supplementary Data 10. Functional annotation of significantly upregulated genes in *Ascl1*-OE tumor cells. DAVID Gene ontology of genes significantly upregulated in *Ascl1*-OE tumor cells using the KEGG and GO categories Biological Processes (BP) and Cellular compartment (CC). Statistical significance of pathway enrichment was determined using Fisher's Exact test.
