# Supplementary material for: Transcription factors ASCL1 and OLIG2 drive glioblastoma initiation and co-regulate tumor cell types and migration
Source: Nat Commun. 2024 Nov 28;15:10363. doi: 10.1038/s41467-024-54750-9 (PMC11605073; doi:10.1038/s41467-024-54750-9)
Supplement: Supplementary file 13 — Reporting Summary [file 41467_2024_54750_MOESM13_ESM.pdf]

nature portfolio

Corresponding author(s): Tou Yia Vue

Last updated by author(s): 9/23/2024

Reporting Summary

Nature Portfolio wishes to improve the reproducibility of the work that we publish. This form provides structure for consistency and transparency in reporting. For further information on Nature Portfolio policies, see our Editorial Policies and the Editorial Policy Checklist.

Statistics

For all statistical analyses, confirm that the following items are present in the figure legend, table legend, main text, or Methods section.

n/a	Confirmed	
☐	☒	The exact sample size (*n*) for each experimental group/condition, given as a discrete number and unit of measurement
☐	☒	A statement on whether measurements were taken from distinct samples or whether the same sample was measured repeatedly
☐	☒	The statistical test(s) used AND whether they are one- or two-sided<br>*Only common tests should be described solely by name; describe more complex techniques in the Methods section.*
☐	☒	A description of all covariates tested
☒	☐	A description of any assumptions or corrections, such as tests of normality and adjustment for multiple comparisons
☐	☒	A full description of the statistical parameters including central tendency (e.g. means) or other basic estimates (e.g. regression coefficient) AND variation (e.g. standard deviation) or associated estimates of uncertainty (e.g. confidence intervals)
☐	☒	For null hypothesis testing, the test statistic (e.g. *F*, *t*, *r*) with confidence intervals, effect sizes, degrees of freedom and *P* value noted<br>*Give P values as exact values whenever suitable.*
☒	☐	For Bayesian analysis, information on the choice of priors and Markov chain Monte Carlo settings
☒	☐	For hierarchical and complex designs, identification of the appropriate level for tests and full reporting of outcomes
☒	☐	Estimates of effect sizes (e.g. Cohen's *d*, Pearson's *r*), indicating how they were calculated

Our web collection on statistics for biologists contains articles on many of the points above.

Software and code

Policy information about availability of computer code

Data collection	No software was used.
Data analysis	Data were demultiplexed and fastq files were generated using bcl2fastq (v2.20.0.422, Illumina) with parameter "--barcode-mismatches" set to 1. Fastq files were aligned and genes/cells were counted against the mouse reference genome (mm10) using CellRanger (v6.0.0, 10xGenomics). Seurat (v5.1.0), an R package (v4.3), was used for downstream unsupervised clustering analyses (https://www.R-project.org/). We used scatter (v1.26.1) to identify and remove cells that were outliers for counts, features, and mitochondrial counts. scDblFinder (v1.12.0) was used to identify and remove doublet cells. Data were transformed using SCTransform (v0.3.5) as implemented in Seurat. Differential gene expression between control and Ascl1-OE tumors for each cell type was determined using FindMarkers in conjunction with the R package MAST (v1.28.0) as implemented in Seurat. Meta module scoring based on previously published GBM cell states was completed using the R package scalop (v1.1.0). All heatmaps of the Cell Type Unionized RNA-seq were generated using the R package ComplexHeatmap (v2.18.0).

For manuscripts utilizing custom algorithms or software that are central to the research but not yet described in published literature, software must be made available to editors and reviewers. We strongly encourage code deposition in a community repository (e.g. GitHub). See the Nature Portfolio guidelines for submitting code & software for further information.

April 2023

Data

Policy information about availability of data

All manuscripts must include a data availability statement. This statement should provide the following information, where applicable:

- Accession codes, unique identifiers, or web links for publicly available datasets
- A description of any restrictions on data availability
- For clinical datasets or third party data, please ensure that the statement adheres to our policy

Single cell RNA-sequencing and ASCL1 and OLIG2 ChIP-sequencing data were deposited into the Gene Expression Omnibus (GEO) database under the accession numbers GSE247650, GSE152401, and GSE247977, respectively. scRNA-seq data and OLIG2 ChIP-seq data are currently private and the ASCL1 ChIP-seq data is publicly available through the following link:
ASCL1 ChIP-seq - https://www.ncbi.nlm.nih.gov/bioproject/639169

Research involving human participants, their data, or biological material

Policy information about studies with human participants or human data. See also policy information about sex, gender (identity/presentation), and sexual orientation and race, ethnicity and racism.

Reporting on sex and gender	N/A
Reporting on race, ethnicity, or other socially relevant groupings	N/A
Population characteristics	N/A
Recruitment	N/A
Ethics oversight	N/A

Note that full information on the approval of the study protocol must also be provided in the manuscript.

Field-specific reporting

Please select the one below that is the best fit for your research. If you are not sure, read the appropriate sections before making your selection.

[x] Life sciences [ ] Behavioural & social sciences [ ] Ecological, evolutionary & environmental sciences

For a reference copy of the document with all sections, see nature.com/documents/nr-reporting-summary-flat.pdf

Life sciences study design

All studies must disclose on these points even when the disclosure is negative.

Sample size	Based on our previous and current studies, a minimum sample size of at least 10 (N=10) for control and experimental groups (at least 3 litters/group) were analyzed at a specific time points for survival . This group size was determine using power analysis to provide 80% power to detect a 20% change in survival at 5% significance level.
Data exclusions	Mice that died of unknown causes, of non-tumor related complications (i.e. hydrocephalus), or with tumors of non-CNS origin which do not express glial lineage markers (SOX10, GFAP, SOX2) were excluded from studies. The presence of outliers was assessed with Grubbs' test using Alpha = 0.05.
Replication	Mouse tumor phenotypes for control and experimental conditions were replicated across multiple litters.
Randomization	Allocation was not random as study utilized different transgenic strains of mice as experimental groups. Covariates included litter effects and sex specific effects. Litter effects were controlled by collected brain tumors for each genotype from at least 3 different litters. Sex specific effects were determined by examining the survival curve within each genotype when split by sex and no significant effect was found.
Blinding	Blinding of experimentalist to tumor conditions or groups were not always possible due to expression of unique tumor reporter proteins (tdTomato or GFP) or the cellular phenotype of tumor cells. Quantification of tumor cellular protein expression level (i.e. ASCL1 and OLIG2) and tumor cell and proliferation were performed using Imaris software to minimize bias.

Reporting for specific materials, systems and methods

We require information from authors about some types of materials, experimental systems and methods used in many studies. Here, indicate whether each material, system or method listed is relevant to your study. If you are not sure if a list item applies to your research, read the appropriate section before selecting a response.

Materials & experimental systems

n/a	Involved in the study
	[x] Antibodies
[x]	Eukaryotic cell lines
[x]	Palaeontology and archaeology
	[x] Animals and other organisms
[x]	Clinical data
[x]	Dual use research of concern
[x]	Plants

Methods

n/a	Involved in the study
	[x] ChIP-seq
[x]	Flow cytometry
[x]	MRI-based neuroimaging

Antibodies

Antibodies used	Guinea Pig anti-ASCL1 Johnson Lab (TX518);Kim et al., 2008<br>Mouse anti-ASCL1 BD Biosciences (556604)<br>Rabbit anti-GFAP Millipore (AB5804)<br>Goat anti-SOX10 R&D Systems (AF2864)<br>Rabbit anti-ASCL1 Abcam (ab211327)<br>Rabbit anti-OLIG2 Millipore (AB9610)<br>Mouse anti-OLIG2 Millipore (MABN50)<br>Rabbit anti-SOX2 Millipore (AB5603)<br>Rat anti-PDGFRa BD Pharmigen (558774)<br>Donkey anti-goat - 488 Invitrogen (A11055)<br>Donkey anti-goat - 568 Invitrogen (A11057)<br>Donkey anti-mouse - 488 Invitrogen (A21202)<br>Donkey anti-mouse - 568 Invitrogen (A10037)<br>Donkey anti-rabbit - 488 Invitrogen (A21206)<br>Donkey anti-rabbit - 647 Invitrogen (A31573)<br>Donkey anti-rat - 647 Invitrogen (A78947)<br>Goat anti-mouse - 488 Invitrogen (A11001)<br>Goat anti-mouse - 568 Invitrogen (A11004)<br>Goat anti-rabbit - 488 Invitrogen (A11008)<br>Goat anti-rabbit - 568 Invitrogen (A11011)<br>Goat anti-rabbit - 647 Invitrogen (A21244)<br>Goat anti-rat - 647 Invitrogen (A21247)
Validation	All antibodies were validated through the manufacturer for use in mouse tissue and the specific procedure of immunohistochemistry.

Animals and other research organisms

Policy information about studies involving animals; ARRIVE guidelines recommended for reporting animal research, and Sex and Gender in Research

Laboratory animals	Mus musculus:<br>R26R-T/T<br>Ascl1-F/F;R26R-T/T<br>Olig2-F/F;R26R-T/T<br>Ascl1-F/F;Olig2-F/F;R26R-T/T<br>R26R-tTA/+;TetO-AIG<br>Analyzed at P30, P60, terminal
Wild animals	N/A
Reporting on sex	Both male and female tumor mice were analyzed.
Field-collected samples	N/A
Ethics oversight	All mouse experiments in this study followed NIH guidelines and a research protocol was approved by the Institutional Animal Care and Use Committee (IACUC) at the University of New Mexico Health Sciences Center and UT Southwestern.

Note that full information on the approval of the study protocol must also be provided in the manuscript.

Plants

Seed stocks	N/A
Novel plant genotypes	N/A
Authentication	N/A

ChIP-seq

Data deposition

- [x] Confirm that both raw and final processed data have been deposited in a public database such as GEO.
- [x] Confirm that you have deposited or provided access to graph files (e.g. BED files) for the called peaks.

Data access links<br>*May remain private before publication.*	ASCL1 ChIP-seq - https://www.ncbi.nlm.nih.gov/bioproject/639169 (GSE152401<br>OLIG2 ChIP-seq - private until publication. (GSE247977)
Files in database submission	GSM4614377_R548_HOT_ASCL1.bw<br>GSM4614378_R548_HOT_Input.bw<br>GSM4614379_R738_HOT_ASCL1.bw<br>GSM4614380_R738_HOT_Input.bw
Genome browser session<br>(e.g. UCSC)	UCSC

Methodology

Replicates	Two independent ChIP-seq experiments were performed using PDX-GBMs (R548 and R738) dissected from brains of NOD-SCID mice following transplantation and subsequent exhibition of neurological symptoms.
Sequencing depth	Approximately 30,000 reads per sample
Antibodies	anti-ASCL1 (Mash1) (BD Biosciences, 556604)<br>anti-OLIG2 (Millipore, AB9610)
Peak calling parameters	The ChIPseq signal enriched regions were identified using the “findPeaks” module available in HOMER software (v.4.7).
Data quality	Low-quality reads and duplicate reads were removed from aligned files using “samtools view -bh-F 0 × 04 -q 10” (v1.2) (Li, 2011) and “Picard MarkDuplicates.jar” (v. 1.131) commands (Picard 2018, Broad Institute, GitHub repository).
Software	De novo motif discovery and analysis were performed using “findMotifsGenome” module available in HOMER software (v.4.7).
